# Stripe charge order and its interaction with Majorana bound states in 2M-WS_2_ topological superconductors

**DOI:** 10.1093/nsr/nwae312

**Published:** 2024-09-05

**Authors:** Xuemin Fan, Xiao-Qi Sun, Penghao Zhu, Yuqiang Fang, Yongkang Ju, Yonghao Yuan, Jingming Yan, Fuqiang Huang, Taylor L Hughes, Peizhe Tang, Qi-Kun Xue, Wei Li

**Affiliations:** State Key Laboratory of Low-Dimensional Quantum Physics, Department of Physics, Tsinghua University, Beijing 100084, China; Frontier Science Center for Quantum Information, Beijing 100084, China; Collaborative Innovation Center of Quantum Matter, Beijing 100084, China; Institute for Condensed Matter Physics and Department of Physics, University of Illinois at Urbana-Champaign, Urbana, IL 61801, USA; Institute for Condensed Matter Physics and Department of Physics, University of Illinois at Urbana-Champaign, Urbana, IL 61801, USA; State Key Laboratory of High Performance Ceramics and Superfine Microstructure, Shanghai Institute of Ceramics, Chinese Academy of Sciences, Shanghai 200050, China; State Key Laboratory of Rare Earth Materials Chemistry and Applications, College of Chemistry and Molecular Engineering, Peking University, Beijing 100871, China; School of Materials Science and Engineering, Beihang University, Beijing 100191, China; State Key Laboratory of Low-Dimensional Quantum Physics, Department of Physics, Tsinghua University, Beijing 100084, China; Frontier Science Center for Quantum Information, Beijing 100084, China; Collaborative Innovation Center of Quantum Matter, Beijing 100084, China; State Key Laboratory of Low-Dimensional Quantum Physics, Department of Physics, Tsinghua University, Beijing 100084, China; Frontier Science Center for Quantum Information, Beijing 100084, China; Collaborative Innovation Center of Quantum Matter, Beijing 100084, China; State Key Laboratory of High Performance Ceramics and Superfine Microstructure, Shanghai Institute of Ceramics, Chinese Academy of Sciences, Shanghai 200050, China; State Key Laboratory of Rare Earth Materials Chemistry and Applications, College of Chemistry and Molecular Engineering, Peking University, Beijing 100871, China; Institute for Condensed Matter Physics and Department of Physics, University of Illinois at Urbana-Champaign, Urbana, IL 61801, USA; School of Materials Science and Engineering, Beihang University, Beijing 100191, China; Max Planck Institute for the Structure and Dynamics of Matter, Center for Free-Electron Laser Science, Hamburg 22761, Germany; State Key Laboratory of Low-Dimensional Quantum Physics, Department of Physics, Tsinghua University, Beijing 100084, China; Frontier Science Center for Quantum Information, Beijing 100084, China; Collaborative Innovation Center of Quantum Matter, Beijing 100084, China; Beijing Academy of Quantum Information Sciences, Beijing 100193, China; Southern University of Science and Technology, Shenzhen 518055, China; State Key Laboratory of Low-Dimensional Quantum Physics, Department of Physics, Tsinghua University, Beijing 100084, China; Frontier Science Center for Quantum Information, Beijing 100084, China; Collaborative Innovation Center of Quantum Matter, Beijing 100084, China

**Keywords:** Majorana bound states, 2M-WS_2_, stripe charge order, topological superconductor

## Abstract

To achieve logic operations via Majorana braiding, positional control of the Majorana bound states (MBSs) must be established. Here we report the observation of a striped surface charge order coexisting with superconductivity and its interaction with the MBS in the topological superconductor 2M-WS_2_, using low-temperature scanning tunneling microscopy. By applying an out-of-plane magnetic field, we observe that MBSs are absent in vortices in the region with stripe order. This is in contrast to adjacent underlaying layers without charge order, where vortex-bound MBSs are observed. Via theoretical simulations, we show that the surface stripe order does not destroy the bulk topology, but it can effectively modify the spatial distribution of MBSs, i.e. it pushes them downward, away from the 2M-WS_2_ surface. Our findings demonstrate that the interplay of charge order and topological superconductivity can potentially be used to tune the positions of MBSs, and to explore new states of matter.

## Teaser

A striped charge order has been discovered in the Fu-Kane topological superconductor 2M-WS2, and its interaction with Majorana bound states (MBS) demonstrates a potential way to manipulate the spatial positions of MBSs.

## INTRODUCTION

Topological materials can host exotic Majorana bound states (MBSs) that obey non-Abelian statistics required for topological quantum computing [1,2]. They can be generated via the combination of electronic topology and superconductivity [3–22]. Evidence of MBSs has been observed in topological insulator-superconductor hybrid structures [5–8,18,19,21]. Possible MBSs have also been reported in the vortex cores of intrinsic Fu-Kane topological superconductors [3], such as FeSe_0.5_Te_0.5_ [14,23], Li_0.84_Fe_0.16_OHFeSe [15], 2M-WS_2_ [16] and LiFeAs [24,25], in which bulk superconductivity coexists with topological surface states. Recently, there has been emerging interest in exotic electronic order in intrinsic topological superconductors. Such systems offer a platform to manipulate the MBS and study the interplay between MBSs and the exotic co-existing order. Indeed, a bulk charge density wave (CDW) in strained LiFeAs can renormalize the bulk electronic band structure without changing its topological properties, and can pin vortices to generate arrays of MBSs [26]. Alternatively, since the MBSs in Fu-Kane superconductors live on the surface, modifications of the surface electronic properties can be used to control the position and distribution of MBSs. Furthermore, it is of fundamental interest to study the interplay between surface electronic order and MBSs. As a first step towards understanding the complex interactions between the MBS and other coexisting electronic order, we report the observation of surface charge order in an intrinsic topological superconductor, 2M-WS_2_ , and expect that it can potentially be used to manipulate the MBS.

## RESULTS AND DISCUSSION

**Figure 1.**
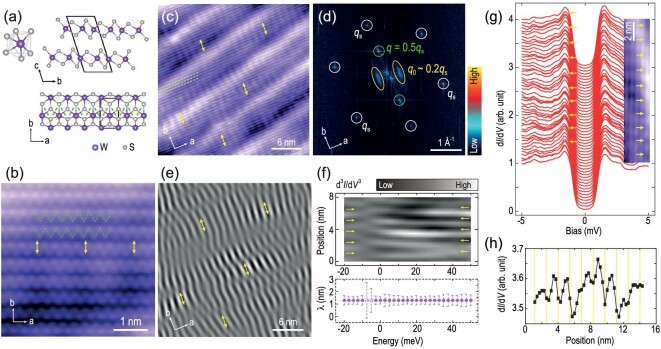
Electronic stripe modulations in 2M-WS_2_. (a) The atomic structure of 2M-WS_2_. Top left panel: schematic of [WS_6_]^8−^ structure. Top right panel: side view of 2M-WS_2_. Bottom panel: top view of 2M-WS_2_. (b) Atomically resolved STM topographic image of WS_2_ (4.4 nm × 4.4 nm; set point, *V*_s_ = −5 mV, *I*_t_ = 300 pA). (c) Stripe modulations over a large area of WS_2_ (30 nm × 30 nm; set point, *V*_s_ = −5 mV, *I*_t_ = 100 pA). (d) Fast Fourier transform (FFT) result of (c). The two parallel line-features (near *q*_0_) correspond to the stripe modulations in real space. The length of *q*_0_ (∼ 0.2*q*_s_) is estimated by the wavevector normal to the line-features. (e) Inverse FFT of *q*_0_. The stripe modulations are extracted and clearly displayed. (f) Top panel: a line cut across several stripes of the second-order derivative of d*I*/d*V* as a function of energy. The yellow arrows denote the valleys of d^3^*I*/d*V*^3^ in this panel. Bottom panel: the period of the stripes as a function of energy, which is determined from the peak position in the FFT result of the d^3^*I*/d*V*^3^ line cut at each energy. The error bar is extracted from the peak width in the FFT. The data labeled with hollow purple circles are in the transition energy ranges, in which the signals are relatively weak. (g, h) Spatially periodic modulations of the superconducting coherence peaks. (g) A series of low-energy d*I*/d*V* spectra (set point, *V*_s_ = −5 mV, *I*_t_ = 400 pA) taken in right inset area. Right inset: STM topography of WS_2_ (2.8 nm × 14.6 nm; set point, *V*_s_ = 10 mV, *I*_t_ = 100 pA). The yellow arrows denote the valleys of the stripe patterns (inset) and the corresponding locations where the spectra are acquired. (h) Position dependence of averaged height of the symmetric coherence peaks in (g), which shows clear periodic spatial modulation. The height of individual coherence peaks is determined by the average of five nearest neighbors near the maximum. The vertical yellow lines correspond to the positions of the yellow arrows in the inset of (g).

We performed low-temperature scanning tunneling microscopy (STM) measurements on 2M-WS_2_ single crystals, in which topological surface states, superconductivity and MBS have been observed [16,27–30]. The material 2M-WS_2_ is a van der Waals crystal constructed from 1T’-WS_2_ monolayers along the *c*-direction (Fig. 1a). Within each monolayer, the deviation of W atoms from the [WS_6_]^8−^ octahedral center leads to a zigzag structure along the *a*-direction (see Fig. 1a). The atomically resolved STM image of WS_2_ (Fig. 1b) shows this zigzag structure (marked by dashed green lines), consistent with the structure in Fig. 1a. The large diagonal features running from bottom left to top right in Fig. 1c correspond to moiré patterns, forming in the cleaving process of the single crystal, during which the topmost layer of WS_2_ is distorted with a small twist angle. The moiré patterns will be discussed in detail later.

In addition to the zigzag atomic chains, we also observe long-range electronic stripe modulations in STM topographic images (denoted by yellow arrows in Fig. 1b and c). These stripes break the rotational and translational symmetries of the lattice and have an incommensurate spatial period of 1.31 nm with slight fluctuations (1.24–1.38 nm) in different regions of the sample. Moreover, in contrast to that of ordinary charge order previously revealed by STM [31,32], the orientation of the stripes observed here is not uniform, and its local distortions in the orientation generate a line-segment-like feature (rather than spot-like feature) centered at the wave vector *q*_0_ in the fast Fourier transform (FFT) image, as shown in Fig. 1d. Note that *q*_s_ and *q* in Fig. 1d correspond to the wave vectors of the S-S lattice and the zigzag chain, respectively, and they still manifest as spot-like features. To highlight the stripe modulations in Fig. 1c, we perform inverse FFT (IFFT) to *q*_0_ and the distorted stripe patterns are clearly shown in Fig. 1e. Scanning tunneling spectroscopy (d*I*/d*V* spectrum) measures the local electronic density of states (DOS), while d*I*/d*V* mapping probes the spatial distribution of DOS at specific energies over a certain area [33,34]. Figure 1f shows the energy-dependent line cut of the stripes extracted from a series of d*I*/d*V* mappings at the same location. The stripe has a π phase shift between the negative and positive energies. Besides, the period of the stripes is unchanged at different energies, indicating that the observed stripes are static electronic modulations rather than quasiparticle interference patterns. Such charge modulation extends to the energy range of -20 meV to 50 meV.

Low-energy-scale d*I*/d*V* spectra taken at 400 mK exhibit U-shaped superconducting gaps, indicating the nodeless superconducting pairing in 2M-WS_2_ (Fig. 1g). Such an observation is consistent with previous work [16]. In addition, the superconducting coherence peaks show spatially periodic modulations. The periodic modulations of the coherence peaks are closely related to the corresponding locations where the spectra are acquired (inset, Fig. 1g). The spectra with lowest superconducting coherence peaks are always taken at the valleys of the stripe patterns (yellow arrows in Fig. 1g). The periodic evolution of averaged heights of the coherence peaks at different locations is summarized in Fig. 1h, demonstrating the intimate correlation of superconductivity and stripe charge order. Applied vertical magnetic fields up to 12 T do not change the distribution of the stripes (Supplementary Material Fig. S1), indicating a non-magnetic origin of the stripes.

**Figure 2.**
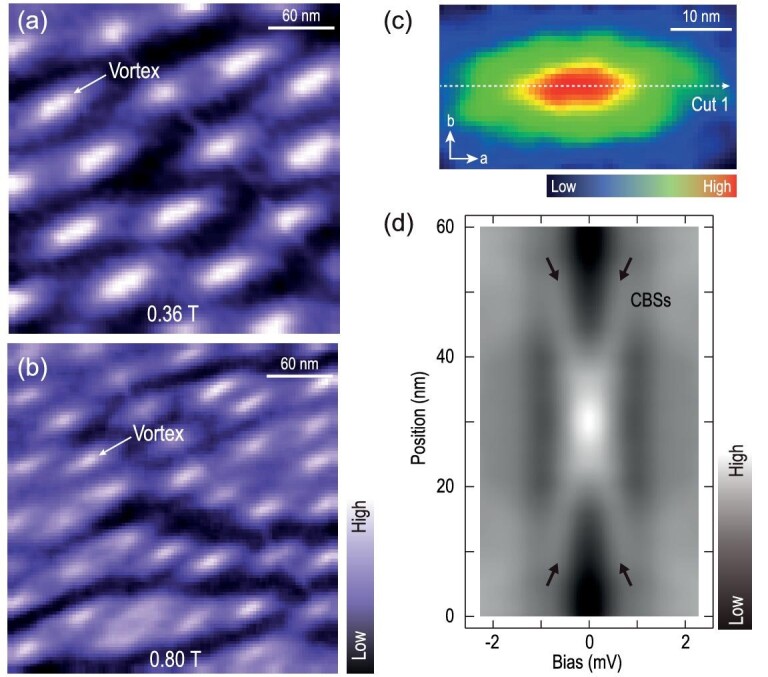
Spatial distribution of the magnetic vortex lattice and absence of a Majorana bound state in the vortex core of WS_2_ with stripes. (a, b) Zero-bias conductance maps of WS_2_ (300 nm × 300 nm; set point, *V*_s_ = −5 mV, *I*_t_ = 200 pA) taken at (a) 0.36 T and (b) 0.8 T, respectively. (c) A zero-bias conductance map (60 nm × 30 nm; set point, *V*_s_ = −5 mV, *I*_t_ = 200 pA) of a single vortex at 0.36 T. (d) False-color image of a series of d*I*/d*V* spectra (set point, *V*_s_ = −4 mV, *I*_t_ = 400 pA) measured along cut 1 in (c). Only CBSs are observed.

Figure 2a and b show the magnetic-field dependence of the vortices. The density of the vortices increases with elevated magnetic field (Fig. 2b). The *H*_c2_ is slightly inhomogeneous over the sample, due to the existence of local strain [35]. The estimation of *H*_c2_ is between 1.6 T and 1.9 T (see Fig. S2).

Bulk 2M-WS_2_ hosts a topologically non-trivial electronic structure [27,36] with a non-trivial *Z*_2_-invariant [28]. Below the superconducting transition temperature (*T*_c_), 2M-WS_2_ without surface charge order becomes a Fu-Kane topological superconductor and has an odd number of the MBSs trapped at the center of vortex cores [16]. Interestingly, in the 2M-WS_2_ with stripe charge modulation observed here, the MBSs are absent in the vortices. To affirm this, we have checked more than 35 vortices in the stripe regions from multiple different samples, and a zero-bias conductance map of a typical single vortex is shown in Fig. 2c; it is elongated along the *a-*direction. A series of d*I*/d*V* spectra shown in the false-color image in Fig. 2d were taken across the vortex along cut 1 of Fig. 2c. The zero-bias conductance peak at the vortex core splits into two symmetric branches in energy as the tip moves away, which can be attributed to Caroli-de Gennes-Matricon bound states (CBSs) [37–39]. Besides the splitting branches, no remaining zero-bias peak coexists with CBSs off the vortex center in the spectra [16], indicating the absence of MBSs in the vortices of 2M-WS_2_ within the stripe region.

**Figure 3.**
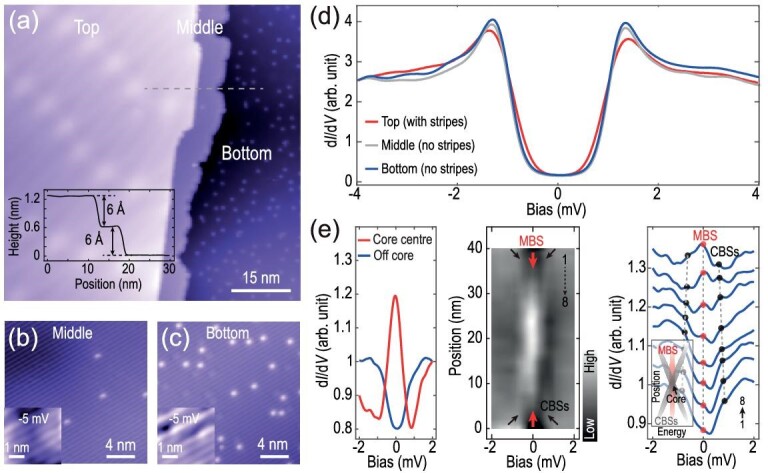
Recovered Majorana bound states in the underlying WS_2_ layer with no stripes. (a) Large-scale topographic image of WS_2_ (75 nm × 75 nm; set point, *V*_s_ = −2 V, *I*_t_ = 20 pA) that includes three layers of WS_2_. The top layer is the region with the stripe patterns. Inset: STM topographic scan profile taken across the two steps, denoted by the gray dashed line in (a), and the step heights are both 6 Å, corresponding to the *c*-axis lattice constant of 2M-WS_2_. (b, c) Topographic images of the middle (20 nm × 20 nm; set point, *V*_s_ = −1 V, *I*_t_ = 50 pA) and bottom (20 nm × 20 nm; set point, *V*_s_ = 1 V, *I*_t_ = 20 pA) layers, respectively. Insets: zigzag chains of S atoms (4 nm × 4 nm; set point, *V*_s_ = −5 mV, *I*_t_ = 300 pA). The stripes are absent in both layers. (d) Averaged d*I*/d*V* spectra of the three layers (set point, *V*_s_ = −4 mV, *I*_t_ = 200 pA). The unaveraged spectra are shown in Fig. S3. (e) A series of d*I*/d*V* spectra taken along the elongated direction (set point, *V*_s_ = −4 mV, *I*_t_ = 200 pA) of a magnetic vortex on the bottom layer at 0.36 T. Left panel: d*I*/d*V* spectra measured on vortex core (red curve) and away from core (blue curve). False-color image of the spectra (middle panel) demonstrates three branches of bound states denoted by red and black arrows in the vicinity of the vortex. The red and black dots on the spectra (right panel) indicate the three-peak feature and the spatial evolutions of the Majorana bound state and CBSs off the vortex core. Note that, due to the energy resolution of our STM, the zero-bias-peak at the vortex core is a mixture of MBS and adjacent CBSs (see Fig. S4). Inset: schematic of the evolution of the coexisting Majorana bound state and CBSs.

We investigated the charge order in different samples and demonstrated that the stripe modulations develop on only the topmost layer and in certain regions of WS_2_. Figure 3a shows an area including three WS_2_ layers, named top layer, middle layer and bottom layer. The step height between two adjacent layers is 6 Å, corresponding to the monolayer thickness of WS_2_. The top layer is the one that we have shown before, in which the moiré pattern and the stripes are observed. The moiré pattern may be formed during the cleaving process. In contrast, the stripe and moiré patterns are absent in the middle and bottom layers (Fig. 3b and c). A small number of adatoms are visible in the underlying layers, which might be the residual alkali metal atoms used during the synthesis of 2M-WS_2_ [27]. These observations suggest that the striped charge modulation is a surface phenomenon and is localized on the surface. Furthermore, superconductivity is slightly enhanced in the underlying layers with no stripes, where stronger coherence peaks and cleaner superconducting gaps are observed in the d*I*/d*V* spectra (Fig. 3d).

Remarkably, the suppression of the stripes and the enhancement of superconductivity allow the MBS to recover in the vortices on the bottom layer. Correspondingly, a zero-bias conductance peak shows up in the d*I*/d*V* spectrum at the vortex center (left panel, Fig. 3e). From the d*I*/d*V* spectra taken across the vortex along the *a*-axis, an apparent three-peak feature appears in the d*I*/d*V* spectra off the core center (right panel, Fig. 3e), which is the same as that in a previous study of MBSs in WS_2_ [16], demonstrating the existence of MBSs. In the false-color image (see the black and red arrows in the middle panel and the schematic in the inset of the right panel of Fig. 3e), the evolution of the splitting branches and the non-split branch are clearly presented. The appearance of non-split zero-bias peak [16,26] indicates the recovered MBS in the vortex in 2M-WS_2_ without stripes.

To emphasize the universality of the observed stripe charge order and its effects on MBSs, we have tested multiple 2M-WS_2_ samples in two separate STM systems and did find consistent results, indicating that the phenomena reported here are not accidental.

**Figure 4.**
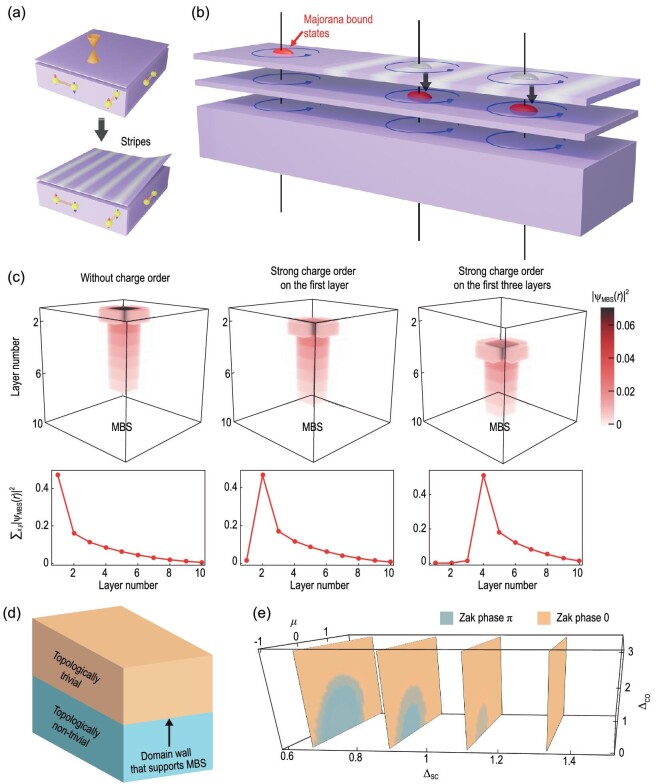
Suppression of Majorana bound states by the surface stripe order. (a) Development of the stripes during the sample cleavage. (b) Suppression of MBSs in the surface stripe region. MBSs are pushed downward to the layer beneath the topmost layer with stripe order. (c) Tight-binding lattice calculation results for the norm-squared of the MBS wavefunctions (${{| {{{{\mathrm{\psi }}}_{MBS}}( {\mathrm{r}} )} |}^2}$) on the top surface without and with strong surface charge order. This quantity corresponds to the probability of finding an electron/hole at different positions. The top three panels show the real space distribution of ${{| {{{{\mathrm{\psi }}}_{MBS}}( {\mathrm{r}} )} |}^2}$ for the MBS, and the bottom three panels show the norm-squared of wavefunctions along the normal direction of the layers, corresponding to ${{}}_{x,y}{{| {{{{\mathrm{\psi }}}_{MBS}}( {\mathrm{r}} )} |}^2}$. Notice that we also include cases of adding charge order to multiple layers near the surface. (d) A domain wall created by adding an extensive number of layers with strong charge order on the top surface. In contrast to other local effects such as a random impurity potential, adding charge order can push the MBS to any depth due to the formation of the domain wall that supports the MBS. (e) Bulk phase diagram in parameter space spanned as a function of the chemical potential *μ* relative to the Fermi-level, the charge order strength Δ_CO_, and the superconductivity pairing strength Δ_SC_ from tight-binding calculations. The details of the tight-binding model and the convention of the energy unit can be found in Methods. The blue region has Zak phase π (topologically non-trivial) and the yellow region has Zak phase 0 (topologically trivial). The two sides of the domain wall highlighted in (d) correspond to the two phases in (e).

Now we discuss the possible origin for the stripe order in 2M-WS_2_ based on our observations. First, the conventional mechanism for charge order induced by the electron–phonon interaction can be excluded. By using density functional theory, we calculate the electronic structures and phonon bands of monolayer, bilayer and bulk 2M-WS_2_. We do not observe any negative phonon mode or phonon softening in these materials with the change of carrier doping (see Figs S5 and S6). Second, we speculate that the cleaving process of the single crystal may play an important role for the development of the stripes and the moiré structures (Fig. 4a). The formation of the moiré superstructures on the topmost layer of WS_2_ may induce local strain and modify the surface electronic structure, giving rise to the development of the stripe order. Stripes and moiré patterns seem to have a symbiotic relationship, although similar stripes appear on the surfaces with different moiré patterns (Fig. 1c and Fig. S1). The moiré patterns look different to each other (Fig. 1c and Fig. S1) due to the varying local strain or twist angles at specific locations. We cannot find the exact correlation between the stripes and the moiré patterns (Fig. S7). Therefore, a conclusion for the origin of the stripes cannot be drawn yet, and further future work is required. Another intriguing property is the distortion of the stripe order mentioned before, which contributes to the decoherence of the long-range order. The possibility of defects acting as possible perturbations of the stripes [40], however, has not been observed in our atomically resolved STM topography (Fig. S8).

Next, we discuss the existence and suppression of MBSs in the surface stripe region. From the Fu-Kane mechanism [3] we expect an MBS to appear in vortex cores when the topological surface states are gapped by the intrinsic superconductivity with a proper bulk chemical potential and pairing strength [11,41]. Tuning these quantities can generate a topological phase transition such that the MBS may disappear (e.g. [41]). In our case, we have observed the MBS on the bottom layer (with no stripes) of 2M-WS_2_, indicating that these conditions for the bulk properties can be fully satisfied. We expect that surface modulations should not locally gap out the vortex-bound MBS when vortices are not overlapping laterally on the surface, but our observations indicate their absence. This seeming contradiction is resolved, once we realize that the MBS does survive in the region with the stripe order, but its profile and position are shifted deeper into the bulk away from the top layer by the charge order (Fig. 4b). Moreover, since the superconducting gap away from the surface is only slightly modified compared to the surface itself (see Fig. 3d) we expect that the driving force for the suppression of MBS is the charge order on the surface. Such phenomena are in contrast to the observation in the strained LiFeAs, in which the strain-induced bulk charge order gaps the bulk states near the *E*_F_ and effectively tunes the chemical potential more favorably for the formation of MBS states in the pinned vortex center [26]. To understand these phenomenological observations, we build a tight-binding lattice model (see Figs S9–11) with the non-trivial topological character, and implement both superconductivity and charge order as the mean field potentials. The numerical simulations of this model in the presence of a flux tube/vortex show that the MBS can be pushed downwards by surface charge order, when its magnitude is greater than a critical value (see Fig. 4c). This is consistent with our experimental observation, i.e. the absence of MBS on the topmost layer with strong stripe order.

Finally, we discuss the significance of the proposed mechanism of modulating the MBS wavefunction via surface charge order, compared with other accidental mechanisms such as local impurity potentials, which were argued as being the reason for the absence of MBSs in intrinsic topological superconductors [14,23,42]. Using our model, we tested the effects of introducing charge order to more layers near the surface and found that, in contrast to the impurity potential scenario, we can control the location of the MBS within a wide range of depths beneath the surface (see Fig. 4c). This can be understood from the limiting case of inducing charge order on a thick bulk layer near the top surface of the material (Fig. 4d) and analyzing the domain wall problem between the two bulk regimes. On the two sides of the domain wall, the bulk flux tube topology can be characterized by a Zak phase calculation (see Supplementary Data), and the resulting detailed phase diagram is shown in Fig. 4e. The MBS is guaranteed to occur at the domain wall if the two sides around the domain wall belong to different topological phases (Fig. 4d). Therefore, the surface charge order observed in our system provides a reliable way to control the MBS's depth by effectively trivializing the surface and creating a domain wall between a topologically trivial phase and non-trivial phase on the flux tube.

## CONCLUSION

In summary, we observed a striped surface charge order coexisting with superconductivity in the topological superconductor 2M-WS_2_. Notably, this charge order influences the positioning of the MBSs, compelling them to relocate beneath the 2M-WS_2_ surface. Our findings not only pave a new path towards manipulating MBSs for quantum information processing and shielding them from spurious external stimuli, but also establish a platform with a rich phase diagram for future studies of complex electronic states in intrinsic topological superconductors.

## METHODS

### Sample preparation and experimental method

To synthesize the precursor K_0.7_WS_2_, the reactants W, S and K_2_S_2_ powders were mixed in a stoichiometric ratio and pressed into a pellet in an argon glove box, which was sealed in an evacuated silica tube at 10^−5^ Torr. The tube was heated up to 850°C for 5°C/min, maintained at this temperature for 6000 minutes, and cooled to 600°C at a rate of 0.1°C/min in the muffle tube. The as-synthesized K_0.7_WS_2_ (0.1 g) crystals were dispersed in de-ionized water and stirred in the acidic K_2_Cr_2_O_7_ (0.01 mol/L) aqueous solution for 1 hour at room temperature. Finally, the 2M-WS_2_ crystals were obtained after washing them in distilled water several times and drying them in the vacuum oven.

Our STM experiments were performed on an ultra-high vacuum (UHV) commercial STM system (Unisoku) which reaches a base temperature of 400 mK by using a single-shot ^3^He cryostat. The base pressure of the system is 2.0 × 10^−10^ Torr. Crystalline WS_2_ samples were cleaved *in situ* at 78 K then transferred into STM. All the measurements were done at 400 mK. A polycrystalline PtIr STM tip was used and calibrated using Ag island before STM experiments. STS data were taken by standard lock-in method. The feedback loop is disrupted during data acquisition and the frequency of the oscillation signal is 973.0 Hz.

## Supplementary Material

nwae312_Supplemental_File
