# Supplementary material for: Stripe charge order and its interaction with Majorana bound states in 2M-WS2 topological superconductors
Source: Natl Sci Rev. 2024 Sep 5;12(2):nwae312. doi: 10.1093/nsr/nwae312 (PMC11745157; doi:10.1093/nsr/nwae312)
Supplement: nwae312_Supplemental_File [file nwae312_supplemental_file.pdf]

Supplemental Material

Stripe charge order and its interaction with Majorana bound states in 2M-WS_2 topological superconductor

Xuemin Fan[1,2,3†], Xiao-Qi Sun[4†], Penghao Zhu[4†], Yuqiang Fang[5,6†], Yongkang Ju[7], Yonghao Yuan[1,2,3], Jingming Yan[1,2,3], Fuqiang Huang[5,6*], Taylor L. Hughes[4], Peizhe Tang[7,8*], Qi-Kun Xue[1,2,3,9,10*] and Wei Li[1,2,3*]

[1]*State Key Laboratory of Low-Dimensional Quantum Physics, Department of Physics, Tsinghua University, Beijing 100084, China*

[2]*Frontier Science Center for Quantum Information, Beijing 100084, China*

[3]*Collaborative Innovation Center of Quantum Matter, Beijing 100084, China*

[4]*Institute for Condensed Matter Physics and Department of Physics, University of Illinois at Urbana-Champaign, Urbana, Illinois 61801, USA*

[5]*State Key Laboratory of High Performance Ceramics and Superfine Microstructure, Shanghai Institute of Ceramics, Chinese Academy of Science, Shanghai 200050, China*

[6]*State Key Laboratory of Rare Earth Materials Chemistry and Applications, College of Chemistry and Molecular Engineering, Peking University, Beijing 100871, China*

[7]*School of Materials Science and Engineering, Beihang University, Beijing 100191 China*

[8]*Max Planck Institute for the Structure and Dynamics of Matter, Center for Free-Electron Laser Science, 22761 Hamburg, Germany*

[9]*Beijing Academy of Quantum Information Sciences, Beijing 100193, China*

[10]*Southern University of Science and Technology, Shenzhen 518055, China*

[†]These authors contributed equally to this work.

[*]To whom correspondence should be addressed: huangfq@mail.sic.ac.cn; peizhet@buaa.edu.cn; qkxue@mail.tsinghua.edu.cn; weili83@tsinghua.edu.cn

I. DISTRIBUTION OF STRIPES UNDER MAGNETIC FIELD

To address the magnetic field dependence of the distorted stripes in 2M-WS_2, differential topographic images of the same area are investigated (Fig. S1) under 0 T and 12 T, respectively. The distributions of the stripes in the two images are identical, exhibiting that the stripes are not sensitive to the external magnetic field.

The monoclinic features in large periodicity in Fig. S1 are moiré patterns.

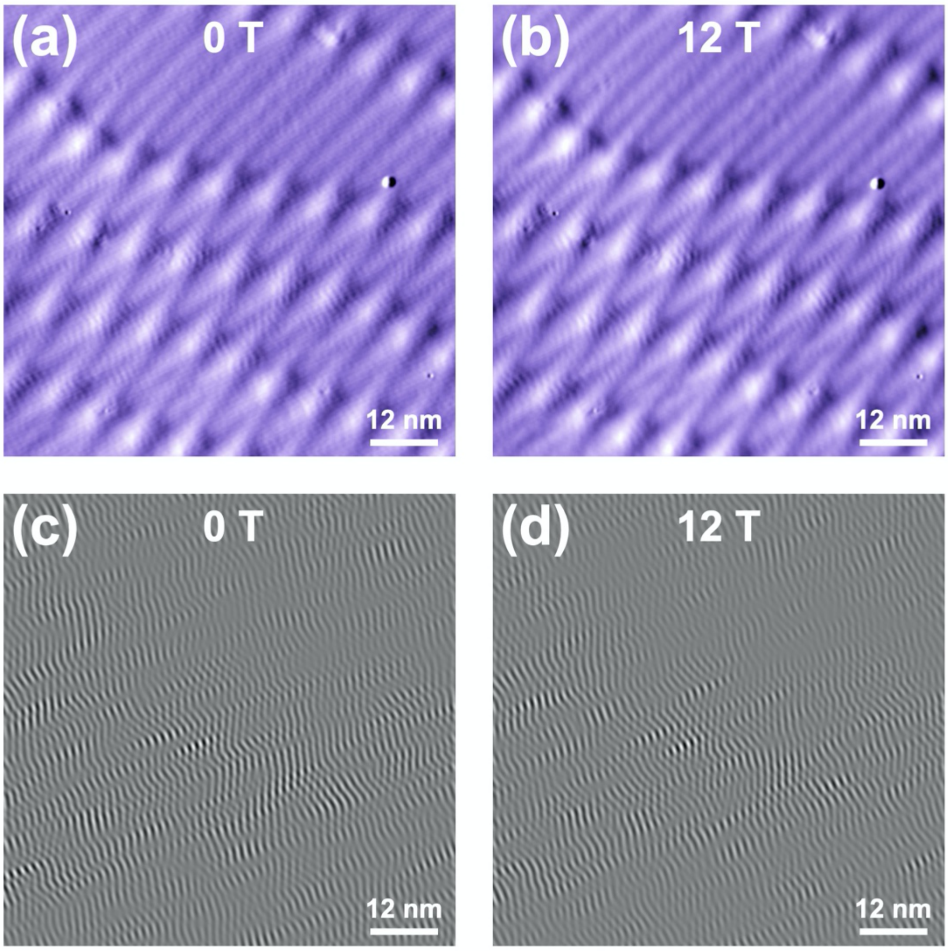

FIG. S1. Robust distribution of stripe modulations under magnetic field. (a)-(b) Derivative along the *x*-axis of a topographic image of WS_2 (80 nm × 80 nm; set point, V_s = -5 mV, I_t = 100 pA) taken at 0 T (a) and 12 T (b), respectively. (c)-(d) Inverse fast Fourier transform result of the stripe-related q_0 calculated from (a) and (b). The distributions of stripes in (a) and (b) are identical to each other, indicating the robustness of the stripes to the magnetic field.

II. ESTIMATION OF H_{C2}

We estimate the H_{C2} by analyzing the tunneling spectra acquired from areas away from vortices under different magnetic fields. Given the inhomogeneity of H_{C2}, we extracted zero-bias conductance (ZBC) in the spectra of two typical areas and summarized in Fig. S2. The linear fitting results suggest that the upper critical field is between 1.6 T to 1.9 T over the whole sample.

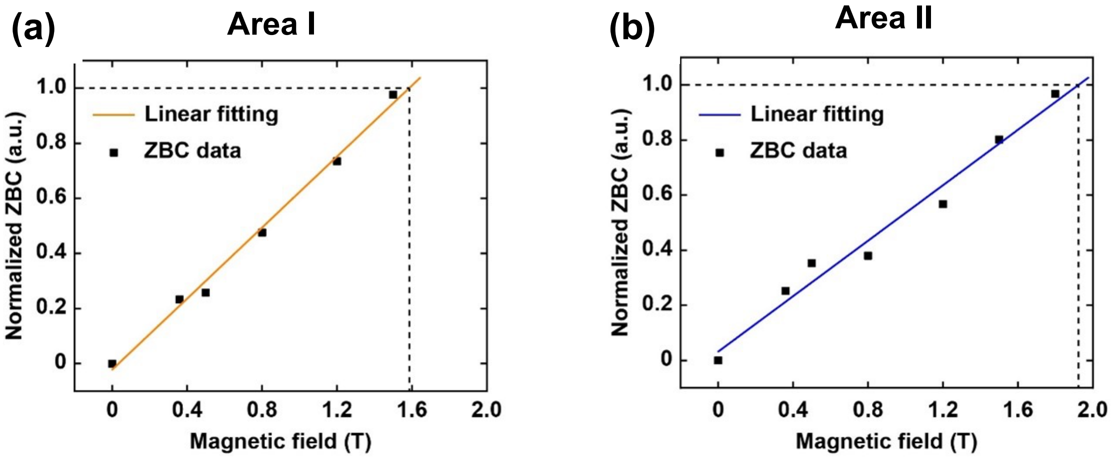

FIG. S2. Estimation of H_{C2} of the sample. (a) Zero-bias conductance (ZBC) extracted from dI/dV spectra taken in Area I (with lower H_{C2}) away from vortices. (b) ZBC extracted from dI/dV spectra taken in Area II (with higher H_{C2}). The spectra are normalized by their dI/dV values at -5 mV. Then ZBC values, which reflect the depth of superconducting gaps, are extracted from the normalized spectra. Linear fittings are applied to the ZBC plots as a function of magnetic field and H_{C2} are estimated by determining where the linear fits reach unity.

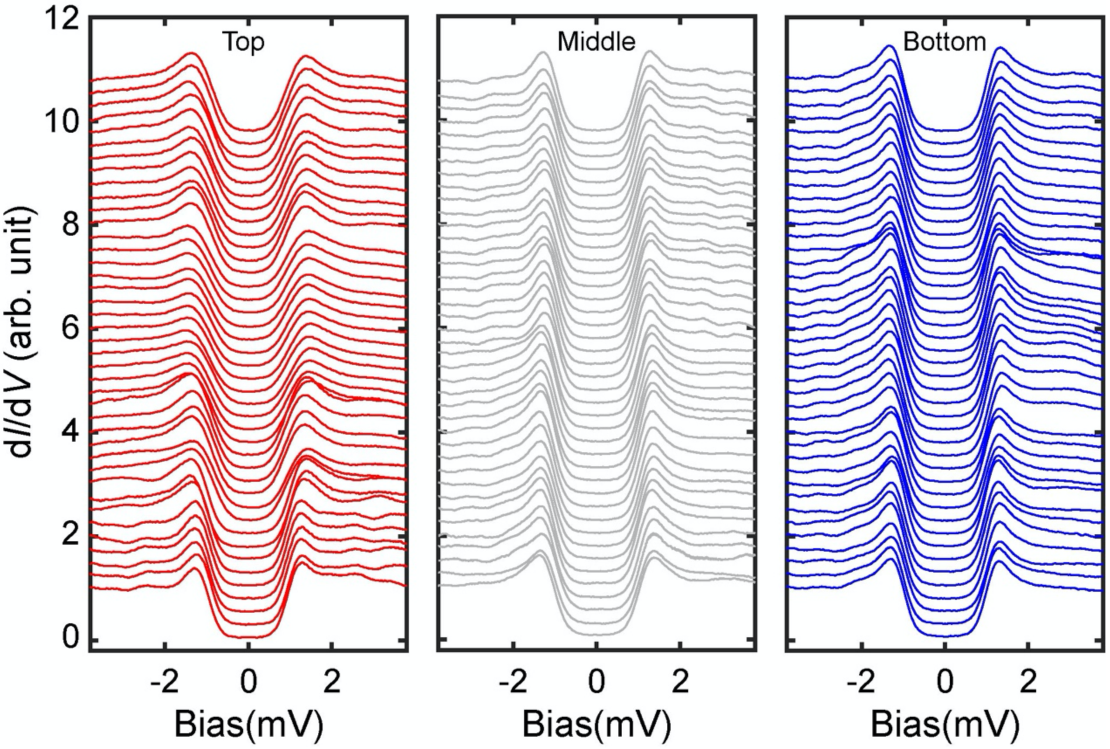

FIG. S3. Superconducting gaps on different layers. Raw dI/dV spectra taken on the top, middle and bottom layers of Fig. 3(a), respectively (set point, V_s = -4 mV, I_t = 200 pA). The difference in superconducting gap size on these three layers is negligible. The coherence peaks on the top layer are slightly lower. The raw dI/dV spectra show consistent results to the averaged spectra in Fig. 3(d).

III. ENERGY SPACING CALCULATIONS BETWEEN THE MBS AND CBSS

The energy levels of CBSs are estimated to be $E_\mu \approx \mu\Delta^2/E_F$, where μ is the half-integer angular momentum quantum number [43], E_F is the Fermi energy, and Δ is the superconducting gap. The wavefunction maximum of a specific state with an angular momentum of μ is located at $r_\mu \approx |\mu|/k_F$ from the vortex core where k_F is the momentum of Fermi surface, giving rise to the splitting peaks away from the vortex core. The energy spacing $\Delta E \approx \Delta^2/E_F$ is usually smaller than the energy resolution of STM.

In 2M-WS_2, the Fermi level across not only the topological surface band, but also the bulk band. The schematic of the MBS and CBSs was shown in Fig. S4. For the topological surface state, the value of the Fermi energy $E_{Fsurface}$ equals to the energy difference between the Dirac point and the Fermi level, which is around -17.9 meV [28]. The superconducting gap is around 1.5 meV for our sample [Fig. 1(g)]. At the vortex cores, quasi-particles are quantized with integer angular momentum quantum number [44], $E \approx \mu_1\Delta^2/E_{Fsurface}$, among which the zero-mode state ($\mu_1 = 0$) is the Majorana bound state. Thus, the energy spacing $\Delta E \approx \Delta^2/E_{Fsurface}$ is 0.126 meV [Fig. S4]. Regarding the bulk band, the value of the Fermi energy E_{Fbulk} equals to the energy difference between the top of the valence band and the Fermi level, which is around -84 meV [28]. The energy spacing between the CBSs originated from the bulk band is $\Delta E_{CBS} \approx \Delta^2/E_{Fbulk} \approx 0.027$ meV. And the energy spacing between the MZM and the adjacent CBSs is $\Delta E' \approx \Delta^2/2E_{Fbulk} \approx 0.013$ meV. Both ΔE_{CBS} and $\Delta E'$ are much smaller than our STM energy resolution (~0.1 meV). Thus, each of the observed splitting peaks in the spectrum is actually an envelope curve of several CBSs. And the spatial non-split branch of zero-bias conductance peak is an experimental criterion for the observation of Majorana bound state [5, 14, 16, 24].

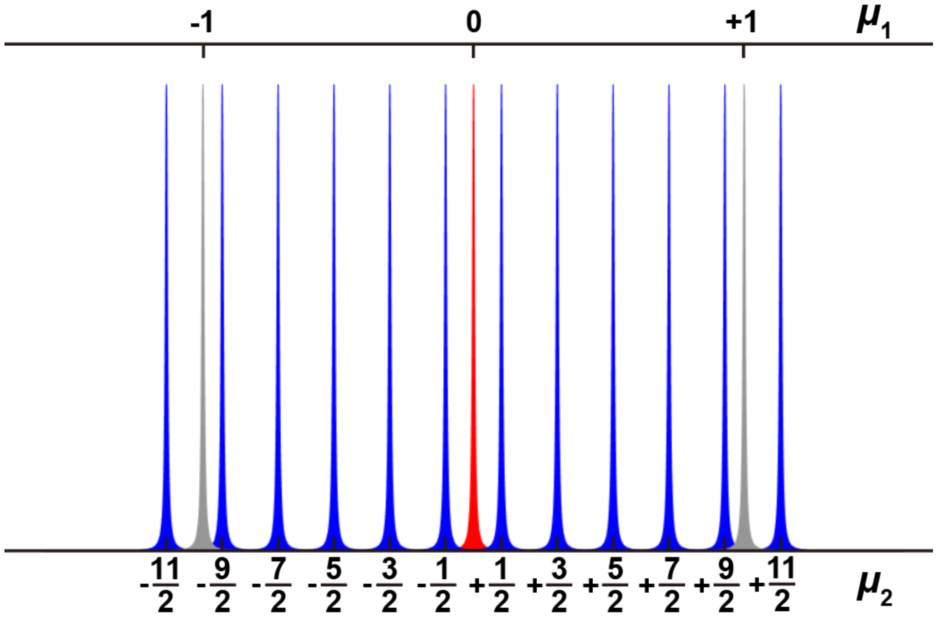

FIG. S4. Schematic of the MBS and CBSs. The black axes mark the eigenvalues of vortex planar angular momenta μ_1 and μ_2, which correspond to the topological surface band and the bulk band, respectively. The gray peaks are the CBSs originated from the topological surface

band, which have integer values of μ_1. The red peak corresponds to the MBS. The blue peaks are the CBSs originated from the bulk band, which possess half-odd-integer angular momenta μ_2.

IV. FIRST-PRINCIPLES CALCULATIONS

We performed *ab initio* DFT calculations to obtain the electronic band structures and phonon dispersions for monolayer, bilayer and bulk 2M-WS_2. The electronic band structures are shown in Fig. S5. Our results are consistent with the previous studies[16, 36]. The bulk 2M-WS_2 is topologically non-trivial with inverted band structures around the Γ point. Figure 6 shows the calculated phonon spectrums under three doping conditions, neutral, electron doped and hole doped for monolayer, bilayer, and bulk 2M-WS_2, respectively. The red dashed lines mark the wave vector q_0 for the stripe charge order (approximately 0.48 $Å^{-1}$ along the direction of zig-zag chains) observed in STM. As shown in Fig. S6, we do not observe the softening of any phonon modes around q_0. Therefore, the electron-phonon interaction cannot be the origin of the stripe order.

First-principles calculations were performed with the Vienna *ab initio* simulation package (VASP). The projector augmented wave (PAW) was used to describe the core level of atomic potential[45, 46]. The generalized gradient approximation (GGA) developed by Perdew-Burke-Ernzerhof[47] was used for the exchange-correlation functional. A plane-wave cutoff of 450 eV was used for the wavefunctions. For the self-consistent electronic structure calculations, we set the energy convergence criterion as 10^{-6} eV. The bulk lattice structures were fully relaxed until the force is smaller than 0.001 eV/Å. For the monolayer and bilayer structures, a vacuum layer of 20 Å were adopted along the *z* axis in the supercell to avoid the coupling between neighboring supercells. The phonon band structures for monolayer, bilayer and bulk 2M-WS_2 were calculated by using the PHONOPY package[48]. The spin−orbit coupling effect was included in these calculations for electronic structures, but was absent for the phonon calculations. For the calculations, the Brillouin zones (BZs) were sampled by16 × 8 × 1 k-grid for monolayer and bilayer and 8 × 8 × 10 for bulk. For phonon calculations, we adopted 4 × 2 × 1 supercells for monolayer and bilayer 2M-WS_2 thin film and 2 × 2 × 4 for bulk 2M-WS_2.

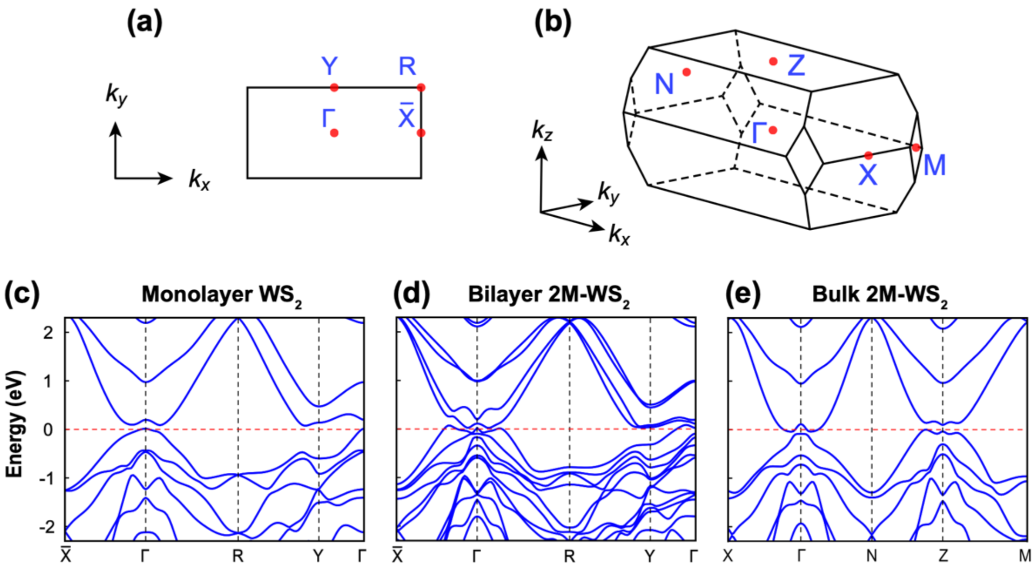

FIG. S5. (a) The Brillouin zone (BZ) of monolayer and bilayer 2M-WS_2. (b) The BZ of bulk

$2M\text{-}WS_2$. (c)-(e) Calculated electronic band structures of the monolayer, bilayer, and bulk $2M\text{-}WS_2$, respectively. The spin-orbit coupling effect is included in the calculation. The red dashed lines denote the Fermi levels.

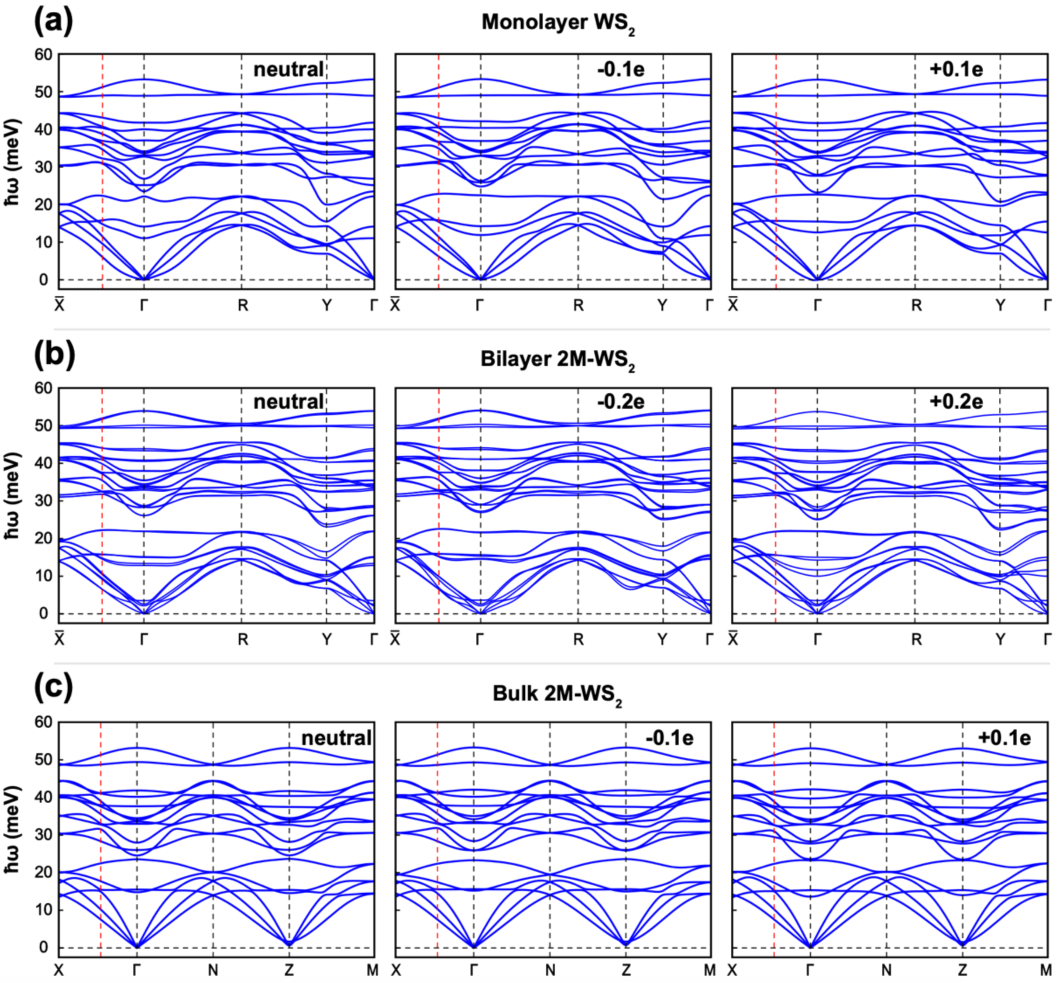

FIG. S6. (a)-(c) The phonon dispersions of monolayer, bilayer and bulk $2M\text{-}WS_2$. Three doping conditions, including neutral, electron doped and hole doped, are considered. The doping concentrations (charge per unit cell) are marked in each plot. For the electron (hole) doping, the value of electrons (holes) doped to each layer is kept to be same for monolayer, bilayer and bulk $2M\text{-}WS_2$. For example, there are two layers in the one-unit cell of bilayer $2M\text{-}WS_2$, thus the doped carriers in bilayer $2M\text{-}WS_2$ per unit cell is twice than that in one-unit cell of monolayer WS_2. The red dashed lines denote the wave vector (q_0) of the observed stripe order. No softening of any phonon mode around q_0 is observed, indicating that the electron-phonon interaction cannot be the origin of the stripe order.

V. THE CROSS-CORRELATION ANALYSIS OF THE STRIPES AND MOIRÉ PATTERNS

A recent study on ZrTe3 has shown that local impurities are able to apply strong pinning potential and thus introduce distortion and phase modulation to CDW[40]. Since the moiré patterns and distorted stripe charge order coexist in WS_2, one natural question would be whether the moiré patterns play the similar role. The following cross-correlation analysis excludes such scenario.

We first extract the stripe and moiré distribution from the topographic image (Fig. S7). Figure S7(b) presents the stripe distribution obtained by taking the inverse fast Fourier transform (IFFT) of the stripe wave vectors in the FFT of Fig. S7(a). Figure S7(c) is the AA sites of moiré patterns in Fig. S7(a), which is obtained by collecting the maximum DOS value in Fig. S7(a). The two images are normalized by the following formula:

$$F(x,y) = \frac{f(x,y) - \mu}{\sqrt{n}\sigma}$$

Here, $F(x,y)$ is the normalized distribution, $f(x,y)$ is the raw distribution, n is the pixel number of f, μ and σ are the mean value and standard deviation of f, respectively.

Then the normalized cross-correlation is calculated using the formula below

$$[F * G](x,y) = \sum\nolimits_{x_0,y_0} F(x_0,y_0)G(x_0+x,y_0+y)$$

Here, $[F * G](x,y)$ is the cross-correlation between F and G with displacement of (x,y). Figure S7(d) shows the cross-correlation result between Fig. S7(b) and (c). Here we zoom-in the displacement to (±10 nm, ±10 nm) region, in the order of single moiré unit cell. The direct onsite influence from the AA site on stripes is easily excluded since the cross-correlation at (0 nm, 0 nm) displacement is negligible. The non-local influence, from long-range interaction or AB site, is also excluded for two reasons: (1) the maximum value in Fig. S7(d) is very small, only about 0.02. This indicates the influence from finite displacement is also negligible. For clarity, a line profile taken along the black arrow is shown in Fig. S7(e) to present the absolute cross-correlation value. (2) The same cross-correlation analysis to another topographic image [Fig. S7(f)] shows a totally different distribution, which indicates the local maxima in the cross-correlation are just from random fluctuations rather than the exact interactions between moiré pattern and stripes.

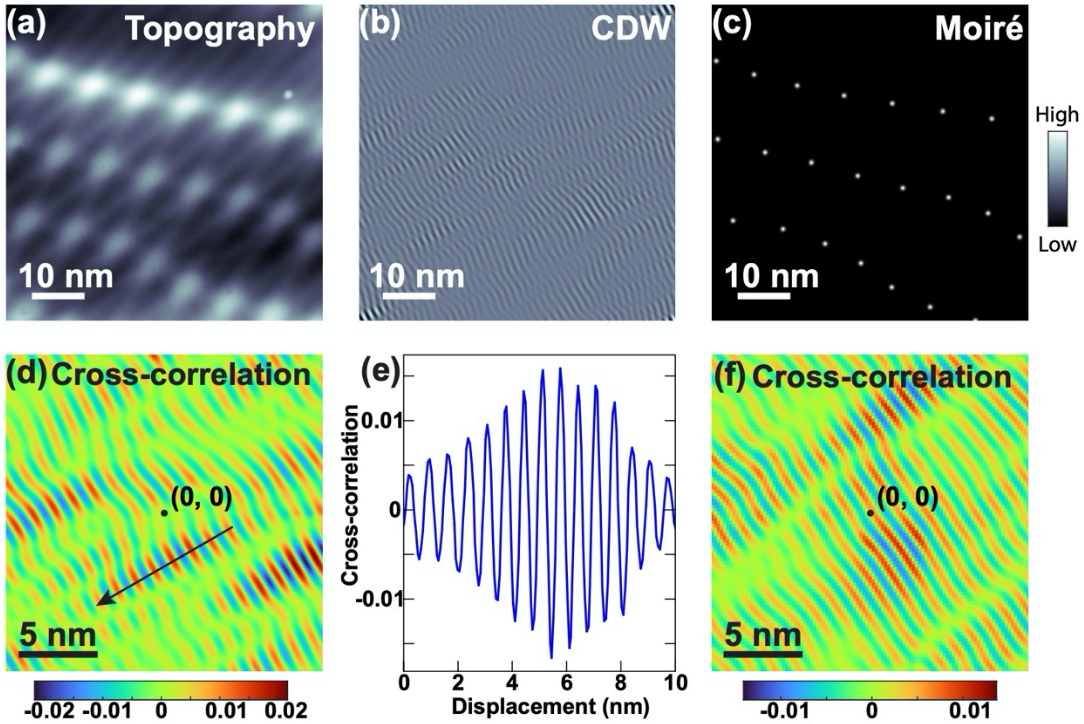

FIG. S7. The correlation between the stripes and the moiré patterns. (a) Typical topographic image of WS_2 with stripes and moiré patterns (set points: V_b = -5 mV, I_s = 100 pA). (b) The stripes map obtained by the IFFT of the stripe wave vectors in the FFT of (a). (c) AA site distribution of the Moiré patterns in (a), which is obtained by collecting the maximum local DOS value in (a). (d) Normalized cross-correlation between the stripes (b) and moiré patterns (c). (e) A line profile along the black arrow in (d). (f) Same cross-correlation analysis to another topographic image, which shows a different distribution.

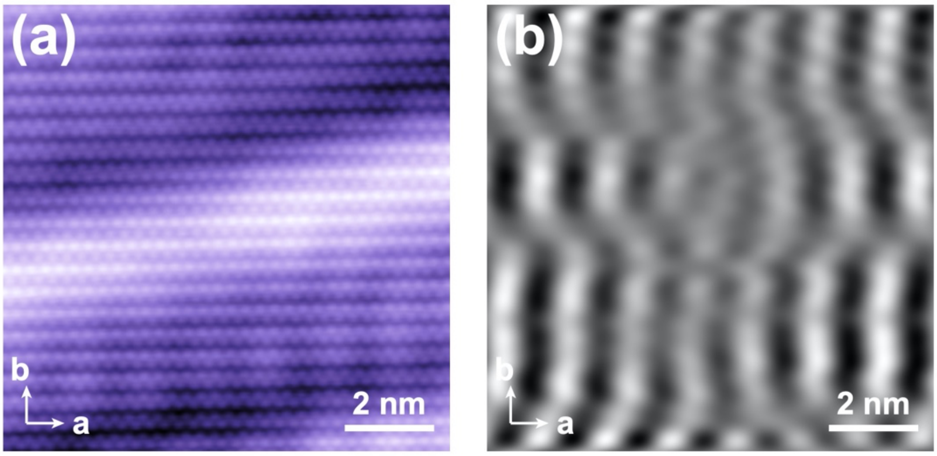

FIG. S8. Defects free region with distorted stripe modulations. (a) Atomically resolved topographic image of WS_2 (10 nm × 10 nm; set point, V_s = -5 mV, I_t = 300 pA) shows a defect-free region with stripe modulations. (b) Inverse fast Fourier transform result of the stripe-related q_0 calculated from a. The stripes are locally distorted in orientation.

VI. TIGHT-BINDING CALCULATIONS

To capture the nontrivial topology and the resulting MBS on the surface of 2M-WS_2, we can study a simple Fu-Kane type tight-binding model[3], which is a 3D topological insulator (TI) in a cubic lattice with s-wave superconducting pairing. We conduct calculations for such a tight-binding model to show that (i) the surface charge order can indeed suppress the MBS from the top surface, and (ii) the suppression of the MBS in position space can be understood from the bulk flux tube topology with the bulk superconductivity and nontrivial topological surface states.

The Bogoliubov-de-Gennes (BdG) Hamiltonian of our tight-binding model is

$$H_{\mathrm{BdG}} = \frac{1}{2}\sum_{k} \Psi_{\mathbf{k}}^{\dagger}[t \sin k_x \xi_z \tau_z \sigma_x + t \sin k_y \xi_z \tau_z \sigma_y + t \sin k_z \xi_z \tau_y \sigma_0$$

$$+(m + \sum_{i=x,y,z} t \cos k_i)\xi_z \tau_x \sigma_0 + (V(\mathbf{k}) - \mu)\xi_z \tau_0 \sigma_0 + \Delta_{SC}(\cos\phi\, \xi_x \tau_0 \sigma_0 + \sin\phi\, \xi_y \tau_0 \sigma_0)]\Psi_{\mathrm{k}}$$

where $k_{x,y,z}$ are the momenta along x, y, z directions, $\xi_{x,y,z}$ are the Pauli matrices for the particle and hole degree of freedom, $\tau_{x,y,z}$ are Pauli matrices for the atomic orbital, and $\sigma_{x,y,z}$ are Pauli matrices for the spin. $\xi_0, \tau_0,$ and σ_0 are 2×2 identity matrices. The electrons are considered to be living on discrete atomic sites and the t is the hopping strength among them. For simplicity, we set $t = 1$ in the following. μ is the chemical potential. $\Delta_{\mathrm{SC}} = \Delta_0 e^{i\phi}$ is the s-wave superconductivity pairing parameter. m is the mass term that controls the topology of the insulating ground state in the absence of the superconductivity and charge order potential: i.e., if we take $-3 < m < -1$, then without superconductivity pairing and charge order potential, the model is a 3D TI that has one surface Dirac cone on each surface. $\Psi_{\mathbf{k}}$ is the Nambu basis that takes the form

$$\Psi_{\mathbf{k}} = \left(c_{\mathbf{k},1\uparrow}\, c_{\mathbf{k},1\downarrow}\, c_{\mathbf{k},2\uparrow}\, c_{\mathbf{k},2\downarrow}\, c_{-\mathbf{k},1\downarrow}^{\dagger} - c_{-\mathbf{k},1\uparrow}^{\dagger}\, c_{-\mathbf{k},2\downarrow}^{\dagger} - c_{-\mathbf{k},2\uparrow}^{\dagger}\right)^{T}$$

Where 1 and 2 (up and down arrows) label orbital (spin) degrees of freedom. $\mathrm{V}(\mathbf{k})$ is a momentum-dependent potential function. With zero $\mathrm{V}(\mathbf{k})$, the model is insulating in the bulk, and with nonzero $\mathrm{V}(\mathbf{k})$, the model generally has no direct gap in the energy spectrum, which is more closed to the experimental situations. However, since the bulk Fermi surface does not qualitatively affect the physics (topology) related to the existence and distributions of MBS, we set $\mathrm{V}(\mathbf{k}) = 0$ for results shown in the main text, and relegate the calculations with nonzero $\mathrm{V}(\mathbf{k})$ in Fig. S9.

To directly observe charge-order induced suppression of MBS from the top surface, we Fourier transform the above Bloch Hamiltonian into real space and calculate the eigenstate wavefunctions for a $10 \times 10 \times 10$ lattice with open boundary conditions along all three directions. We introduce a vortex in the *x*-*y* plane (i.e., a flux tube along the *z*-direction) by assigning different phases of pairing parameters, ϕ, in different patches of the lattice, as illustrated in Fig. S10.

We label the ten discrete atomic layers in our tight-binding model along *z* direction by

$z = 1,2,\dots,10$, where $z = 1$ corresponds to the top layer and $z = 10$ corresponds to the bottom layer. We add the surface charge order potential on the layer at $z = 1$ as $\Delta_{\text{CO}} \cos(Qx)\delta_{z,1}\xi_z\tau_0\sigma_0$, where $\delta_{z,1}$ is the Kronecker Delta function. When Δ_{CO} is large enough, we can observe the suppression of MBS from the top surface to the deeper region as discussed in the main text. Without loss of generality, in all our calculations, we use $m = -2t$, $\Delta_0 = t$, and $Q = 2\pi/3$. The choice of Q will not influence the suppression of MBS from the top surface induced by the surface charge order (see Fig. S11). For the plots shown in Fig. 4(a), we fix $\mu = 0$, and choose $\Delta_{\text{CO}} = 10t$ ($\Delta_{\text{CO}} = 0$) for the second and third (first) panel. Since we are doing calculations in a finite system, there are no exact zero modes - MBS localized on opposite surfaces due to the hybridization between each other. We use a symmetric linear combination for two eigenstates, which are closest to zero energy in our calculations. Then we plot the symmetric results as the probability of the MBS localized on the top surface [i.e., the surface at $z = 1$ as shown in Fig. 4(c)].

Interestingly, if we switch on charge order deeper in the bulk, the MBS is suppressed further into the bulk and appears at the interface between regions with and without charge order [see Fig. 4(c)]. Consequently, a global downward displacement of the entire MBS wavefunction can be observed with the appearance of surface charge order on the top layers [see Fig. 4(c)]. This is because, with the Nambu basis and considering the superconducting pairing, the charge order can lead to a bulk topological phase transition in the 1D flux tube [see Fig. 4(e)], and the interface between regions with and without charge order is a domain wall between distinct topological phases. At the interface [see Fig. 4(d)], the MBS is trapped. Technically, the bulk topological property for each region on opposite sides of the domain wall [marked as blue and yellow in Fig. 4(d)] could be captured by the Zak phase along *z*-direction (i.e., the direction of the flux tube) computed in the translationally invariant bulk, respectively. Conventionally, a system with Zak phase $\pi(0)$ is identified as topologically nontrivial (trivial), which hosts (no) MBS on its boundary. In order to calculate the Zak phase, instead of having all three directions open, we need to keep the *z*-direction periodic so that the momentum along the *z*-direction, k_z, is a good quantum number. Furthermore, our model has a mirror-*z* symmetry ($M_z = \xi_0\tau_x\sigma_z$) and this simplifies the calculation of Zak phase along *z* direction as the product of eigenvalues of the mirror-*z* operator for occupied eigenstates at $k_z = 0$ and $k_z = \pi$. Given a chemical potential μ, a charge order parameter Δ_{CO}, and a superconductivity pairing order parameter Δ_{SC}, we can calculate the corresponding Zak phase. By scanning these parameters, we plot the phase diagram in Fig. 4(e).

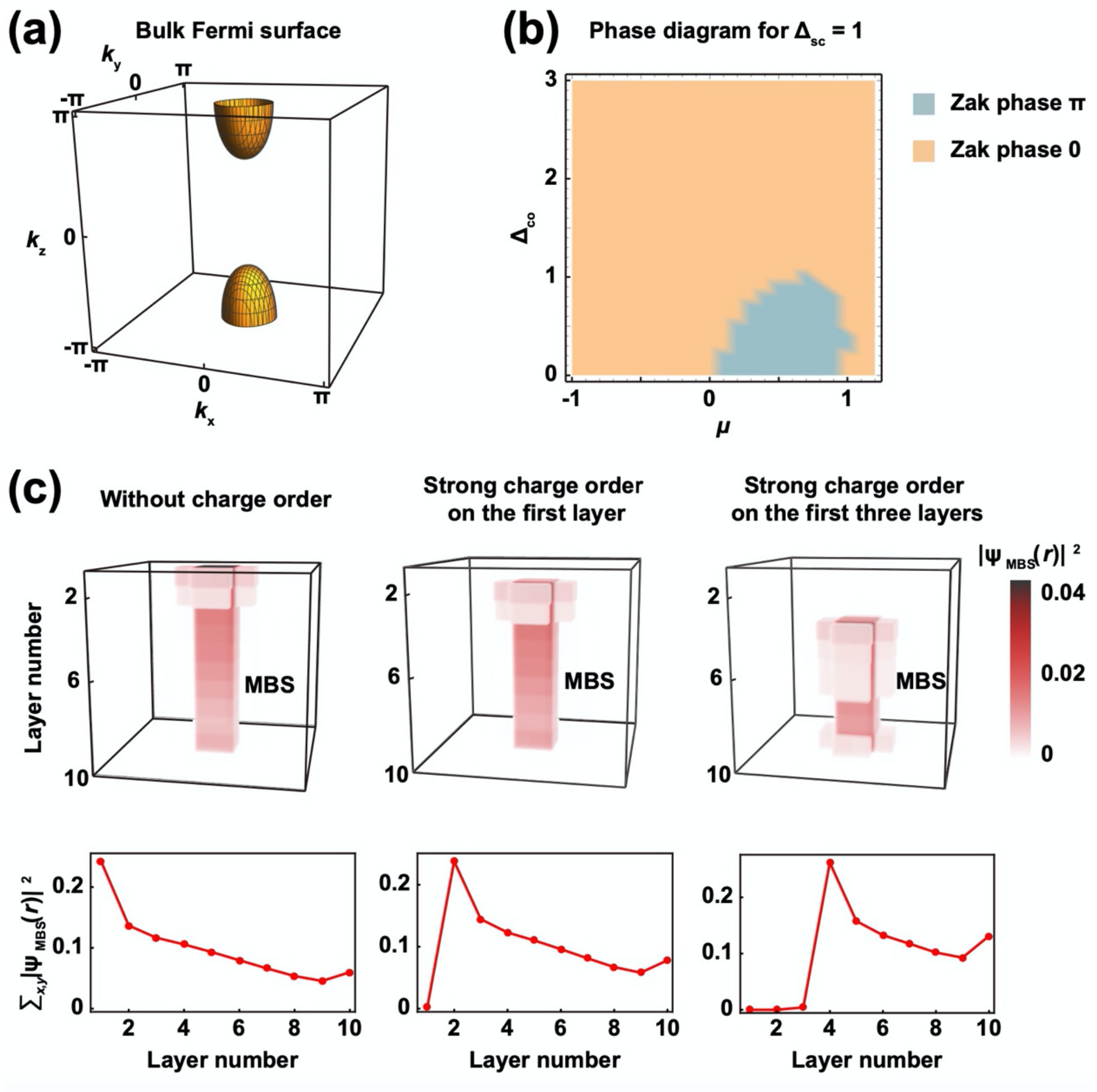

FIG. S9. Phase diagram and Majorana bound states when $V(k) = 1 - 0.5\cos k_z$. (a) Bulk Fermi surface of the tight-binding Hamiltonian. (b) Bulk phase diagram in parameters space spanned by the chemical potential μ, the charge order strength Δ_{CO}, and the superconductivity pairing strength Δ_{SC} from tight-binding calculations. The blue region has Zak phase π (topologically nontrivial) and the yellow region has Zak phase 0 (topologically trivial). (c) Tight-binding lattice calculation results for wavefunctions of MBS on the top surface without and with strong surface charge order. The top three panels show the real space distribution of the wavefunctions for the MBS, and the bottom three panels show wavefunctions along the normal direction of the layers.

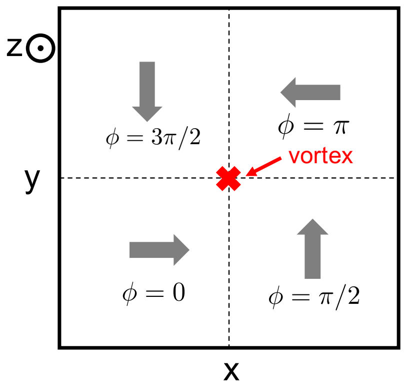

FIG. S10. Implementation of vortex in the *x*-*y* plane. A vortex is implemented by assigning different ϕ for different patches, such that ϕ changes 2π around a loop encircling the red cross. The arrow indicates the direction of the paring parameter in the complex plane.

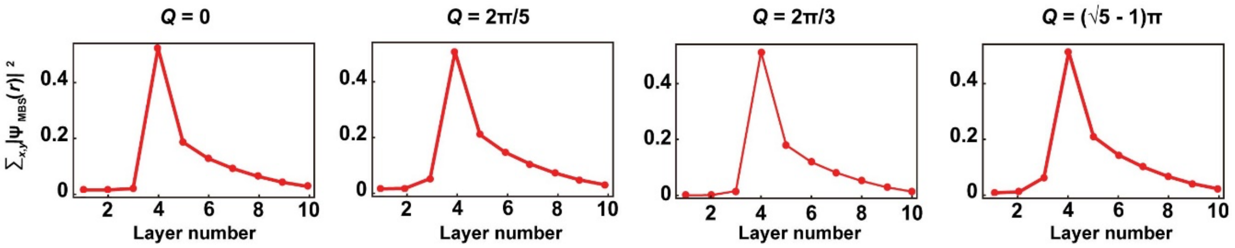

FIG. S11. The layered dependent distribution for MBS with the charge order and different Q value. These plots show the norm-squared of wavefunctions along the normal direction of the layers, corresponding to $\sum_{x,y}|\psi_{MBS}(\mathrm{r})|^2$. The change order is added to the top layer and the value of Q changes.

VII. THE ZERO-BIAS CONDUCTANCE MAP ON THE BOTTOM LAYER

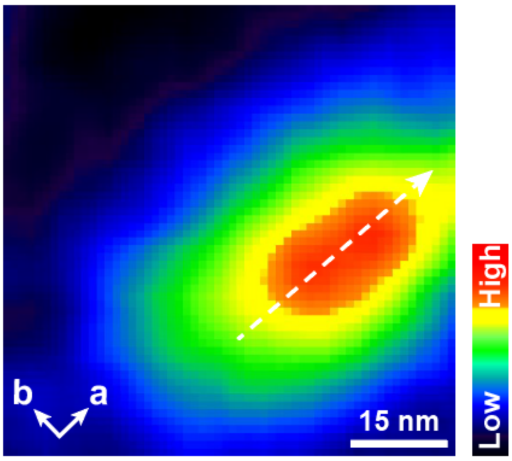

FIG. S12. Spatial distribution of the magnetic vortex on the bottom layer. A zero-bias conductance map (70 nm × 70 nm; set point, V_s = -10 mV, I_t = 100 pA) of the vortex at 0.36 T. The series of dI/dV spectra in the middle panel of Fig. 3(e) was taken along the white dashed line.

[43] Hayashi N, Isoshima T and Ichioka M *et al*. Low-Lying Quasiparticle Excitations around a Vortex Core in Quantum Limit. *Phys Rev Lett* 1998; **80**: 2921-2924.

[44] Stone M and Chung S-B. Fusion rules and vortices in p_x+ip_y superconductors. *Phys Rev B* 2006; **73**: 014505.

[45] Blöchl PE. Projector augmented-wave method. *Phys Rev B* 1994; **50**: 17953-79.

[46] Kresse G and Joubert D. From ultrasoft pseudopotentials to the projector augmented-wave method. *Phys Rev B* 1999; **59**: 1758-75.

[47] Perdew JP, Burke K and Ernzerhof M. Generalized Gradient Approximation Made Simple. *Phys Rev Lett* 1996; **77**: 3865-8.

[48] Togo A and Tanaka I. First principles phonon calculations in materials science. *Scr Mater* 2015; **108**: 1-5.
